# Utilization of temporal autoencoder for semi-supervised intracranial EEG clustering and classification

**DOI:** 10.1038/s41598-023-27978-6

**Published:** 2023-01-13

**Authors:** Petr Nejedly, Vaclav Kremen, Kamila Lepkova, Filip Mivalt, Vladimir Sladky, Tereza Pridalova, Filip Plesinger, Pavel Jurak, Martin Pail, Milan Brazdil, Petr Klimes, Gregory Worrell

**Affiliations:** 1 1St Department of Neurology, Faculty of Medicine, Masaryk University, Brno, Czech Republic; 2 Institute of Scientific Instruments, The Czech Academy of Sciences, Brno, Czech Republic; 3 Department of Neurology, Mayo Clinic, Mayo Systems Electrophysiology Laboratory, Rochester, MN USA; 4 Czech Institute of Informatics, Robotics, and Cybernetics, Czech Technical University in Prague, Prague, Czech Republic; 5 Faculty of Biomedical Engineering, Czech Technical University in Prague, Kladno, Czech Republic; 6 Faculty of Electrical Engineering and Communication, Brno University of Technology, Brno, Czech Republic; 7 International Clinical Research Center, St. Anne’s University Hospital, Brno, Czech Republic; 8 CEITEC – Central European Institute of Technology, Masaryk University, Brno, Czech Republic

**Keywords:** Machine learning, Epilepsy, Computational neuroscience

## Abstract

Manual visual review, annotation and categorization of electroencephalography (EEG) is a time-consuming task that is often associated with human bias and requires trained electrophysiology experts with specific domain knowledge. This challenge is now compounded by development of measurement technologies and devices allowing large-scale heterogeneous, multi-channel recordings spanning multiple brain regions over days, weeks. Currently, supervised deep-learning techniques were shown to be an effective tool for analyzing big data sets, including EEG. However, the most significant caveat in training the supervised deep-learning models in a clinical research setting is the lack of adequate gold-standard annotations created by electrophysiology experts. Here, we propose a semi-supervised machine learning technique that utilizes deep-learning methods with a minimal amount of gold-standard labels. The method utilizes a temporal autoencoder for dimensionality reduction and a small number of the expert-provided gold-standard labels used for kernel density estimating (KDE) maps. We used data from electrophysiological intracranial EEG (iEEG) recordings acquired in two hospitals with different recording systems across 39 patients to validate the method. The method achieved iEEG classification (Pathologic vs. Normal vs. Artifacts) results with an area under the receiver operating characteristic (AUROC) scores of 0.862 ± 0.037, 0.879 ± 0.042, and area under the precision-recall curve (AUPRC) scores of 0.740 ± 0.740, 0.714 ± 0.042. This demonstrates that semi-supervised methods can provide acceptable results while requiring only 100 gold-standard data samples in each classification category. Subsequently, we deployed the technique to 12 novel patients in a pseudo-prospective framework for detecting Interictal epileptiform discharges (IEDs). We show that the proposed temporal autoencoder was able to generalize to novel patients while achieving AUROC of 0.877 ± 0.067 and AUPRC of 0.705 ± 0.154.

## Introduction

Epilepsy is a common neurological disease affecting approximately 50–60 million people worldwide^1^. Even with access to a wide array of anti-seizure medications, about one third of people with epilepsy have drug-resistant epilepsy (DRE) and continue to have seizures^2,3^. Epilepsy surgery with resection of the brain region generating seizures is a treatment option for some patients^4^. Intracranial EEG (iEEG) recording is an essential tool for the localization of epileptic seizure onset zones prior to resection surgery. Modern diagnostic approaches allow continuous iEEG monitoring for numerous days or weeks. The iEEG data size, signal complexity, and quality are increasing due to advanced acquisition systems with higher sampling rates (e.g. 25 kHz) and number of recording channels (e.g., 12 electrodes, each with 12–15 contacts). Spatial sampling across multiple brain structures creates large-scale heterogeneous patient-specific data sets. These novel recording capabilities necessitates automatizing iEEG signal analysis, storage^5^, visualization^6^, classification, and clustering.

Many patients are not suitable resective surgery candidates, due to multifocal epilepsy or seizure onset localized in the eloquent cortex. Electrical brain stimulation is a FDA approved therapy for patients unable to undergo resection surgery^7–9^. Novel implantable devices capable of both sensing and electrical stimulation are emerging at the cutting edge of neuromodulatory treatments for epilepsy^10–12^ and comorbidities^13^. However, even with modern devices capable of continuous sensing (months to years in patients implanted with sensing devices), optimizing closed-loop neuromodulatory treatment is challenging and critically depends on accurate, automated classification of normal and pathological electrophysiological recordings.

Making clinical sense of large complex data sets requires trained electrophysiology experts and it is not possible without spending a substantial amount of time reviewing and annotating the iEEG datasets. It is nearly impossible without automated tools. For instance, the precise localization of pathological tissue in the brain is essential to target surgical and electrical stimulation therapy for epilepsy patients. It has been shown that the level of agreement in interpretation between experts varies and is biased by the subjective experience of the clinician^14–16^. Manual labeling can often create bias and inaccurate gold standards, which results in machine learning systems that don't perform well, because they learned from incorrectly labeled or biased data. Therefore, a manual review of iEEG is expensive, subjective, and time-consuming. It is not sustainable as an approach that can scale up to the amount of iEEG data generated by currently available clinical and research systems.

Recently, machine learning (ML), and deep learning (DL) has become a state-of-the art tool for classification due to its efficacy when trained on big labeled datasets. The power of DL in signal and image recognition is well established^17^, including biological applications, e.g. ECG classification^18^ or transcriptomics^19^. A subclass of deep neural networks, a convolutional neural network (CNN), has been widely applied in signal processing, including EEG analysis^20–22^, sleep scoring^23^, and polysomnography^24^. The main advantage of these methods is that they find optimal features and connections without relying on manual feature engineering^25^. Recently, we and others have shown that supervised deep learning methods can be utilized for iEEG classification. For example, deep neural networks were shown to allow seizure forecasting in humans with neurostimulation devices^26^, scalp EEG^27^, and canine epilepsy models^28^ . However, excellent model performance requires large, accurately labeled datasets. This means a large portion of data must be scored by an expert(s) (electrophysiologist).

Large-scale databases with ground truth (gold-standard) labels are still rare in iEEG. To overcome this issue, we propose using semi-supervised ML technique that enables exploiting features from large-scale unlabeled EEG datasets, with subsequent class assignments based on small amounts of expert gold standard labels. Here, we design and describe an unsupervised autoencoder that is aware of temporal context. The essential key of the method is projecting time series data points into a low-dimensional embedding space. For this purpose, we utilize a neural network autoencoder with a self-attention mechanism as a pre-processing step to perform a dimensionality reduction.. This allows an iterative approach where the technology assists the domain expert, preprocess data, and suggests samples to review to refine class boundaries based on newly provided expert gold standard labels (Active learning). All this aims to speed the learning and analysis up and to create better and more reliable models.

We propose this method to help to automate review of biomarkers in iEEG presurgical monitorings or for big EEG data from continuous iEEG sensing in neurostimulation trials^10,11^. This still remains challenging and it is an active area of clinical and basic research. In general, the aim of this method is in localizing artifacts and abnormal epileptic activity such as interictal epileptiform discharges (IEDs)^29–31^ or high-frequency oscillations (HFOs)^32–35^. In order to support the results of the method, we further provide its pseudo-prospective application that was based on the clinical use case of automated IEDs detection.

## Methods

### Data

In this research, we utilize the publicly available iEEG dataset that was published by our group^36^. We recommend checking the manuscript that describes the data in genuine detail. There, we thoroughly discuss the labeling process, data verification, and estimation of reviewer agreement. Furthemore, the following text provides a brief description of the data to help to understand its utilization within the scope of this work.

**Figure 1:**
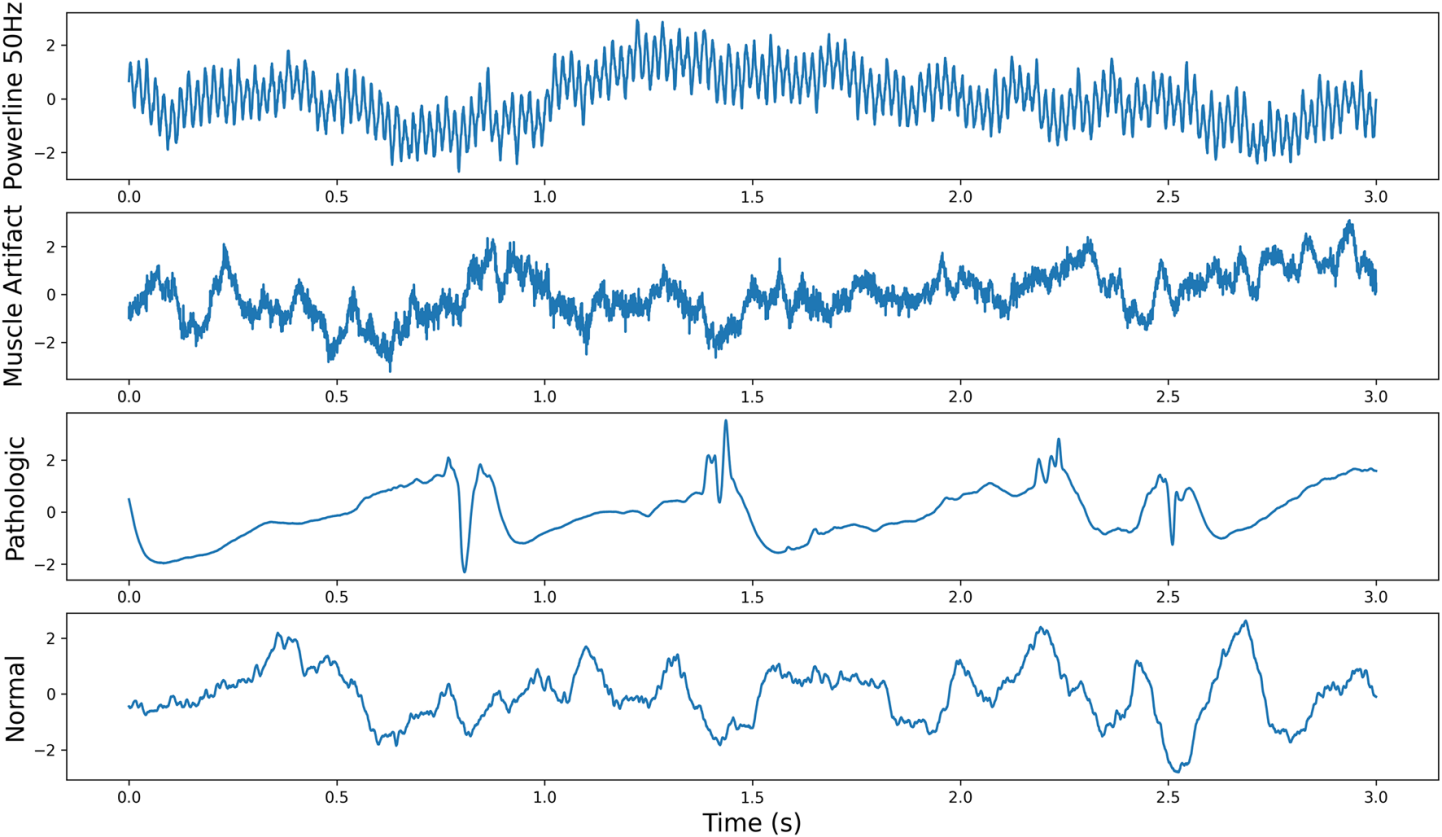
Example of iEEG segments from each classification group in the dataset.

Three independent reviewers manually annotated signals using the power distribution matrix technique (PDM), where each annotated mark was checked in the raw data domain^32^. Visual inspection and manual data annotation were performed in SignalPlant software—free software used for signal post-processing, annotation, and examination^6^. First, signals were filtered by a band-pass filter to highlight high-frequency activity. Then, the PDM approach applied the Hilbert transform on filtered signals to obtain the signal power envelope. A high power envelope was detected in signals and subsequently visually inspected. Based on visual inspection of EEG graphoelements, the segments were classified into one of four classes (Fig. 1): physiological activity (no epileptic biomarkers and no artifactual signals), pathological activity (containing epileptiform graphoelements, e.g., pathologic HFOs and IEDs), muscle artifacts, and power line noise. The pathological activity segments (IEDs, and HFOs) were extracted mainly from seizure onset zones and irritative zones. The segments are considered pathologic if HFO, IED, or HFO superimposed on an IED are present (e.g., Nejedly et al.^20^ Fig. 1). Finally, classified segments were divided into 3-s segments (15,000 samples); the window duration was empirically estimated based on evidence from our former study^21^.

**Figure 2:**
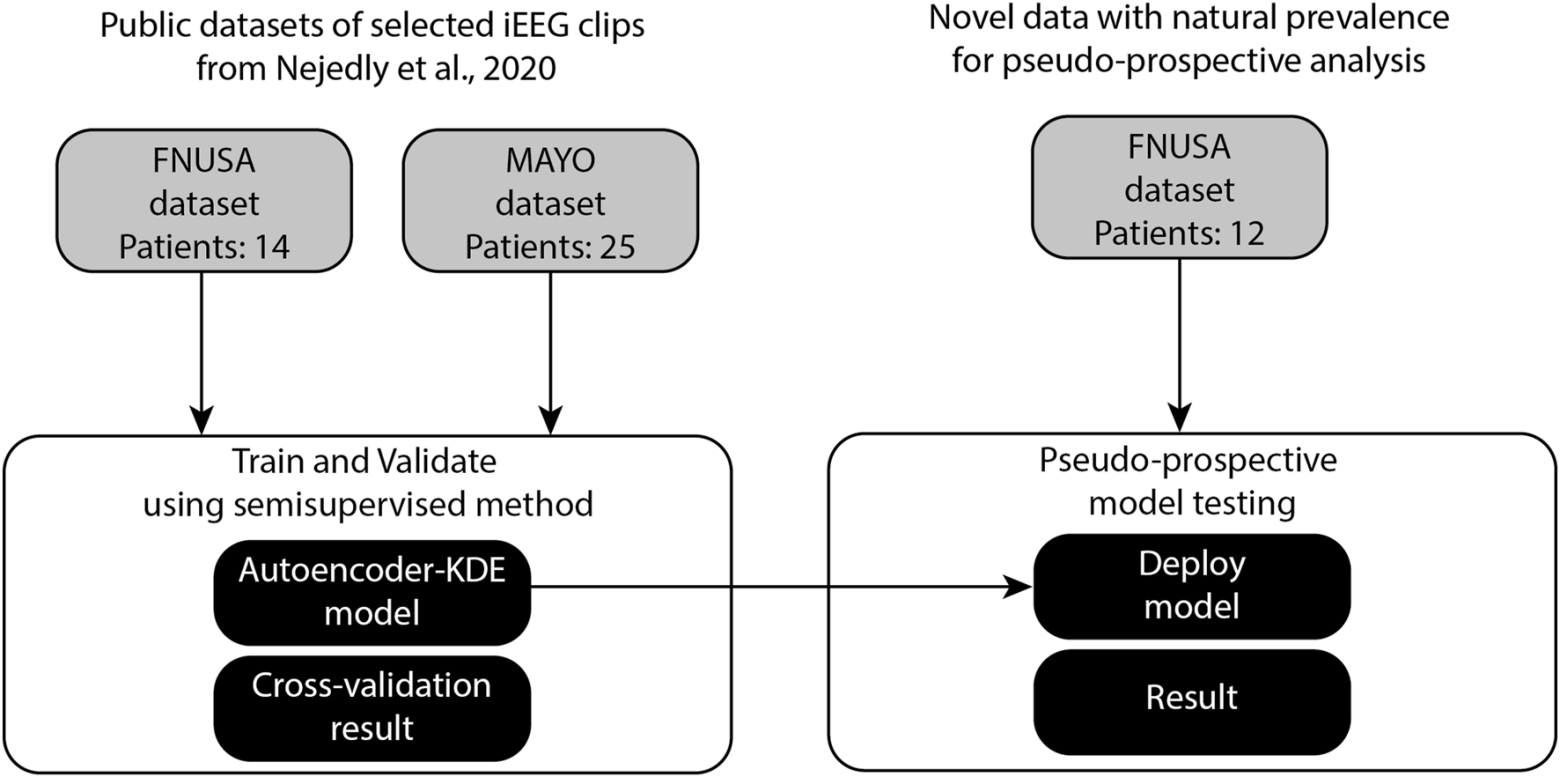
Description of the dataset used in this study. The datasets from Nejedly et al. 2020 were used for model training and validation. Novel data from 12 patients from FNUSA hospital were used for pseudo-prospective testing.

In order to test the generalizability of our approach, we also include 12 novel patients with drug-resistant epilepsy undergoing pre-surgery evaluation from St. Anne’s University Hospital (FNUSA) that were used for pseudo-prospective testing (Fig. 2). One channel of iEEG recording (30 min while awake and resting, which is standard protocol for HFO evaluation^32^ at FNUSA) was selected and manually annotated for IEDs. The data for pseudo-prospective analysis is used in its entire length, which provides a real-world testing benchmark where IEDs have their natural prevalence. No preprocessing for artifact rejection or removal was employed in this data. The proposed test shows a model performance on established clinical protocol^32^.

### St. Anne’s University Hospital dataset

The St. Anne’s University Hospital (FNUSA) dataset was derived from 14 (public dataset)^36^ and 12 (Table 1 ,pseudo-prospective test) patients who underwent pre-surgical iEEG monitoring for DRE. Patients were implanted with standard intracranial depth electrodes (5, 8, 10, 12, 15 and 18 contact semi-flexible multi-contact platinum electrodes (DIXI or ALCIS), with a diameter of 0.8 mm, a contact length of 2 mm, contact surface area of 5.02 mm^2^, and inter-contact distance 1.5 mm). Data was recorded with a custom BrainScope acquisition system (25 kHz sampling frequency; 192 channels). A sampling frequency of 25 kHz was applied during 30 min of recording and subsequently followed by 2 kHz low-pass filtering and 5 kHz down-sampling for further processing and to avoid aliasing. The data are from interictal periods while the patient was awake and resting^32^.

**Table 1:** Clinical description of 12 patients from St. Anne’s University Hospital that were used for pseudo-prospective testing.

Subject	Gender	Age at SEEG	Precipitating event	Age at Seizure onset	MRI before SEEG (signs of)	SEEG monitoring (days)	Type and side of epilepsy	SOZ	Intervention/histopathology	Postoperative outcome Engel (follow-up, year)
1	M	26	–	10	Normal	10	E/RX	Orbitofrontal subgenual cortex	Resection of T pole, subgenual and dorsal part of orbitofrontal cortex	IA (6)
2	M	24	–	14	Lesion (cavernoma) in dorsal part of left cingulate gyrus	12	E/LT	Dorsal part of cingulate gyrus	Lesionectomy/AVM	IB (5)
3	F	27	–	19	Low grade gliom/DNET within posterior part of right cingulate gyrus	10	E/RX	mesial PO area	Lesionectomy/oligodendroglia-like cells	IIIA (5)
4	F	58	–	12	Postsurgical changes (left AMTR)	9	T/bilaterally	Hippocampus bilaterally (mainly right side)	VNS	
5	F	49	–	20	Normal	9	T/RX	Anterior part of GTM, hippocampus	AMTR/negat	IA(5)
6	M	45	Commotio cerebri	16	Left T pole agenesis, arachnoid cyst	10	T/LT	T pole	Cortectomy/FCD III NOS	IA(5)
7	M	28	–	12	suspected FCD in dorsal part of right superior T gyrus	10	T/RX	T pole. superior temporal sulcus	Cortectomy/FCD IIB, nodular heterotophy	IVA (4)
8	M	27	–	10	Normal	8	E/RX	Not founded	VNS	
9	F	25	Meningoencephalitis	5	Postencephalitic changes of left frontal lobe, left hippocampal sclerosis	10	T/LT	Hippocampus	AMTR/HS type I	IA (4)
10	M	47	Commotio cerebri	6	Nonspecific white matter lesions FT bilaterally	9	E/RX	Anterior insula	Cortectomy/FCD Ib	IIIA (4)
11	M	23	–	15	Normal	7	E/LT	Anterior operculoinsular area	Cortectomy/FCD IIa	IC (3)
12	M	27	Commotio cerebri	14	Normal	11	E/LT	Posterior part of lingual gyrus	Cortectomy/negat	IC(3)

*M* male, *F* female; *SEEG* stereoelectroencephalography; *E* extratemporal; *T* temporal; *PO* parieto-occipital; *FT* fronto-temporal; *RX*/*LT* right/left; *GTM* medial temporal gyrus; *SOZ* seizure onset zone; *AMTR* anteromedial temporal resection; *DNET* dysembryoplastic neuroepithelial tumor; *AVM* arteriovenous malformation; *FCD* focal cortical dysplasia; *HS* hippocampal sclerosis; *VNS* vagus nerve stimulation.

### Mayo Clinic dataset

The Mayo Clinic dataset^36^ was derived from 25 patients that underwent pre-surgical iEEG monitoring for DRE. Patients were implanted with stereotactic depth AD-Tech electrodes (AD-Tech Medical Instrument Corp., Racine, WI or PMT, Chahassen, MN, USA) consisting of 4 or 8 Platinum/Iridium contacts (2.3 mm long, 1 mm diameter, spaced 5 or 10 mm center-to-center) and AD-Tech subdural grids and strips electrodes that had 4.0 mm diameter Platinum/Iridium discs (2.3 mm exposed) with 10 mm center-to-center distance. Data was recorded by the Neuralynx Cheetah system (Neuralynx Inc., Bozeman MT, USA; 25 kHz sampling frequency). From the continuous interictal recordings, we selected 2 h long phases (1 AM–3 AM) for our dataset. The data were filtered by a 1 kHz low pass filter and down-sampled to 5 kHz for further processing and analysis.

In total, we used 193,118 and 155,182 3-s iEEG segments from the St. Anne’s University Hospital and Mayo Clinic subjects. Segment distributions for each labeling group are described in Table 2. The dataset consists of four groups: physiological in different behavioral states (wake, wake-relax, sleep), pathophysiological with different biomarkers (IEDs, HFOs), muscle artifacts, and power line noise (50 Hz or 60 Hz depending on the origin of recording—EU/US).

**Table 2:** Labeling categories for iEEG data and the number of samples collected across institutions.

Category	St. Anne’s University Hospital	Mayo Clinic	Reference to results
Physiological activity	94,560	56,730	Class 3
Pathological activity	52,470	15,227	Class 2
Artifacts	32,599	41,303	Class 1
Power line noise (50 Hz/60 Hz)	13,489	41,922	Class 0
Total # of 3-s segments	193,118	155,182	

The full dataset information can be found in^36^.

### Data preprocessing

The iEEG segments were converted into spectrograms by a short-time Fourier transform (STFT) with a window size of 256 samples and a 128 sample overlap. Subsequently, the spectrograms were row and column normalized, forming a three-dimensional tensor [CH, F, T] with CH representing normalization (row and column), F is a spectrogram frequency axis, and T is a spectrogram temporal axis.

### Model architecture

**Figure 3:**
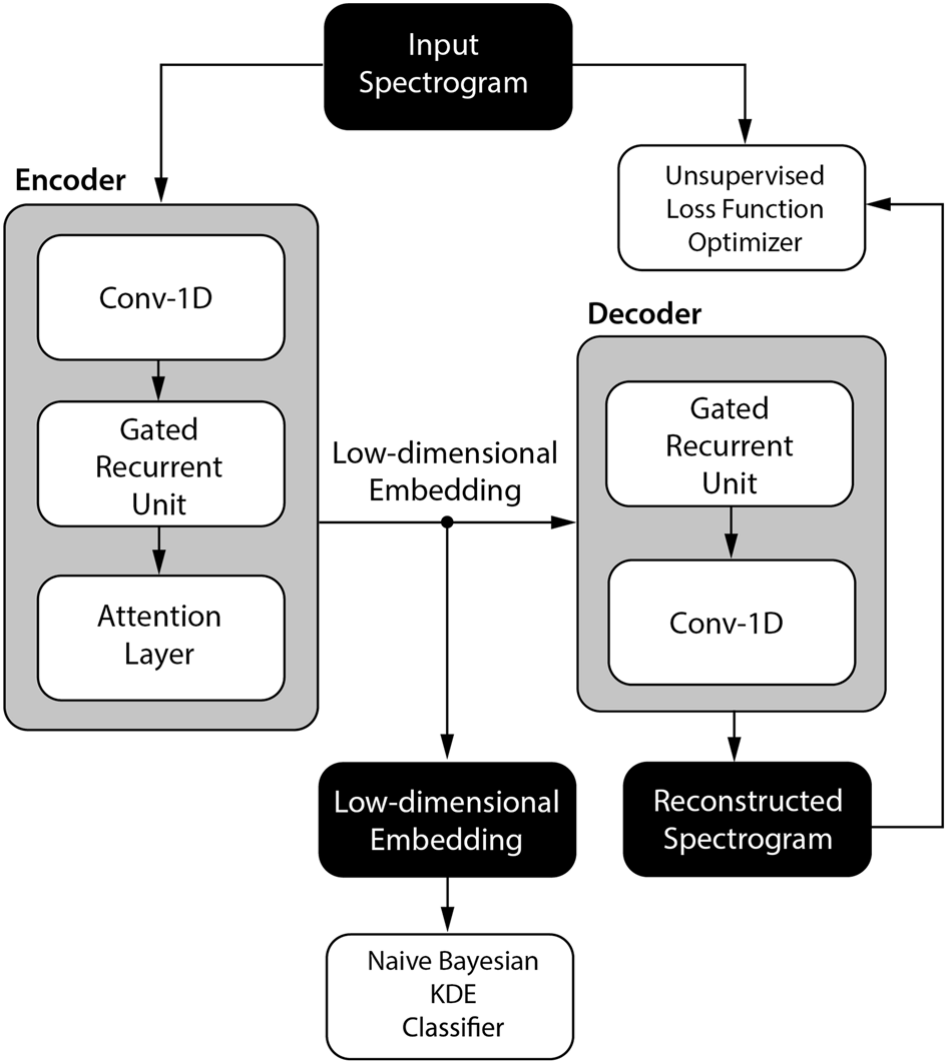
Neural network encoder-decoder architecture. The figure shows a block diagram of neural network encoder-decoder architecture utilizing an attention mechanism for unsupervised iEEG feature extraction. Input consists of iEEG data spectrograms propagated through gated recurrent units (GRU) encoder providing low-dimensional embedding space. Subsequently, the GRU decoder reconstructs the input spectrograms while minimizing the unsupervised loss function (mean square error).

The autoencoder consists of two functional blocks: an encoder and a decoder. The encoder produces low-dimensional embedding space as an output, and the decoder reconstructs the low-dimensional embedding space while minimizing a loss function representing a distance between input and reconstructed output, e.g. mean square error (MSE) or mean absolute error (MAE). Temporal autoencoders are used for time series dimensionality reduction and usually utilize long-short term memory (LSTM) layers or gated recurrent units (GRU) layers that provide low dimensional embedding summarizing the temporal evolution of the input signal. The primary goal of the autoencoder is to learn an input representation mapped in the low dimensional latent space. The autoencoder that is used here (Fig. 3) also utilizes a self-attention mechanism, which improves forgetting problems in encoder-decoder architectures when the model processes long sequences.

### Semi-supervised training workflow

**Figure 4:**
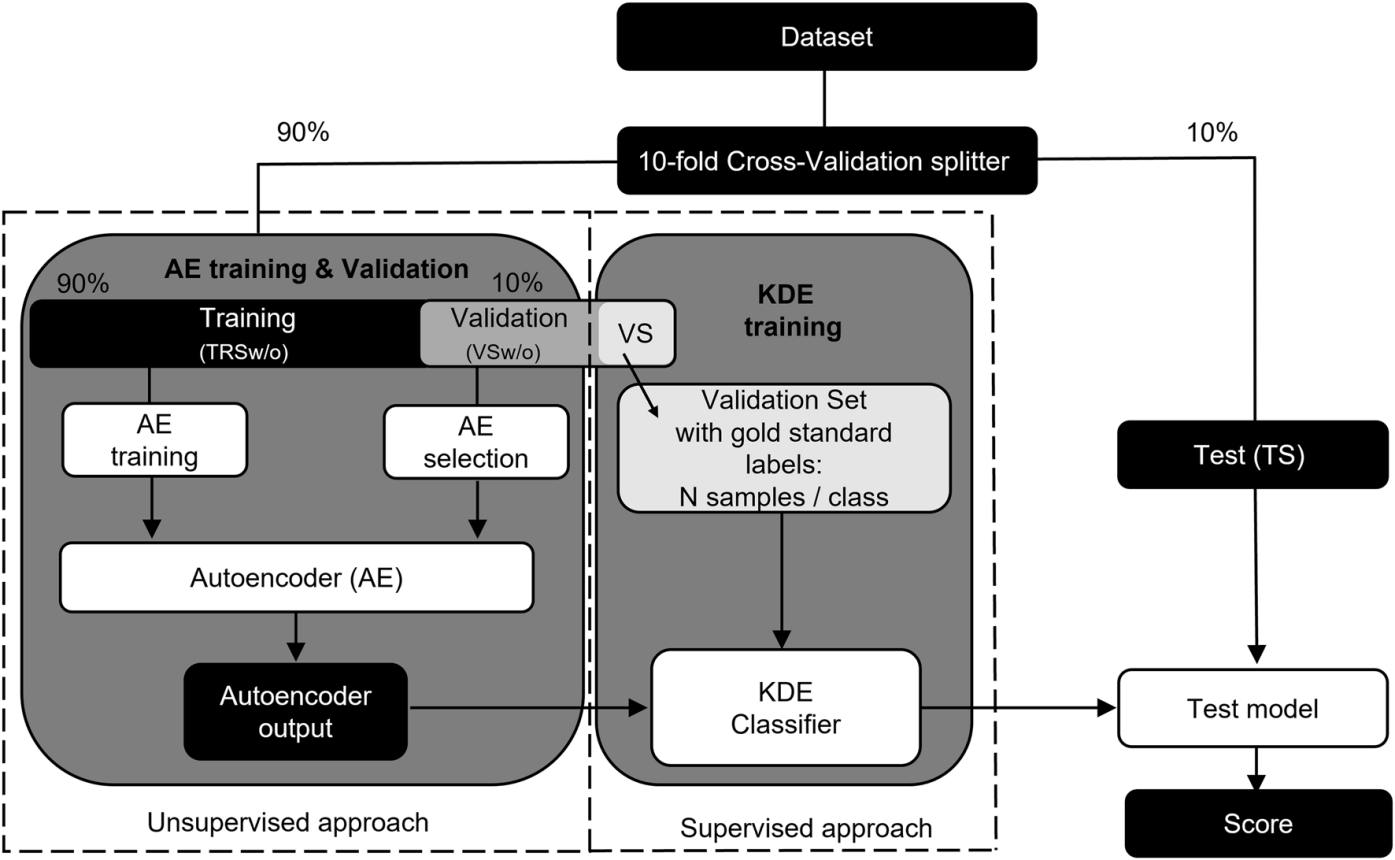
Model training, validation and testing pipeline. The model was tested using tenfold cross-validation methodology.

Before model training, we prepared a tenfold cross-validation testing scheme to provide reliable statistical results (Fig. 4). First, the whole dataset was randomly split into 10 folds, where each fold had the same data label distribution. The 9 folds were used as a training set, and one was used for testing (leave one out cross-validation). Furthermore, the training set was randomly divided into training (90%) and validation (10%). At this point, data labels for the training set were completely discarded, simulating unlabeled data. The validation set used N (experiments with 10, 20, 30, 50, 70, 100, 200, 300, 500, 700, 1000) randomly selected gold-standard samples from each classification group, and the rest of the labels were again completely discarded.

We prepared four datasets: training set without labels (TRSw/o), validation set without labels (VSw/o), a subset of validation subset with N gold standard labels in each group (VS), and testing set (TS) with available gold standard labels. The testing set gold standard labels were used to evaluate model classification performance. This experiment was evaluated for each cross-validation group for each N.

The model was trained for 10 epochs using the training set without labels while minimizing MSE loss function by adaptive moment estimation (ADAM)^37,38^ with learning rate of 10^−3^ and batch size of 128 examples. The reconstruction model performance was monitored by evaluating cosine similarity between input image and reconstruction image on the validation set without labels (data was not used for training). The model with the highest reconstruction performance out of the tenfold training batch was selected as the model for subsequent inference on the test set.

### Kernel density maps estimation

**Figure 5:**
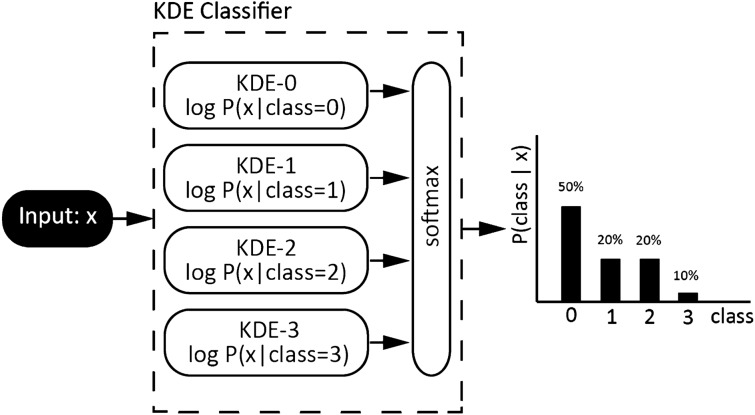
The architecture of the Naive Bayes classifier. The classifier utilizes kernel density estimates (KDE) for prediction of class dependent probability distribution functions. The input to the classifier is the low dimensional embedding extracted from the autoencoder.

Subsequently, for the autoencoder feature extraction (128-dimensional feature space), we employed the kernel density estimation (KDE) technique to estimate the non-parametric probability density function for each class individually (Fig. 5). The validation set with labels (VS, N gold standard samples in each classification group) was used to estimate KDE maps.

### Inference and visualization

The model inference starts with converting the testing samples to the spectrograms, which are processed by the autoencoder providing low-dimensional embeddings. Subsequently, embeddings are compared with the KDE maps, and the class with the highest KDE probability is selected as the model output (Table 3). Such an approach basically simulates the Naive Bayes classifier.

**Table 3:** The confusion matrix for the model shown in the uniform manifold approximation and projection (UMAP) in Fig. 5.

Class	Power line noise (50 Hz/60 Hz)	Artifacts	Pathological activity	Physiological activity
Power line noise (50 Hz/60 Hz)	1348	0	0	0
Artifacts	0	2472	241	547
Pathological activity	0	30	4608	609
Physiological activity	0	131	245	9080

**Figure 6:**
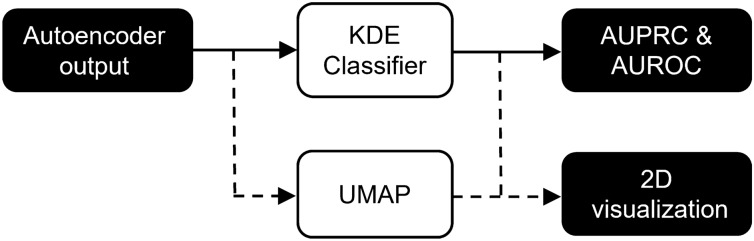
Figure shows pipeline for model inference and uniform manifold approximation and projection (UMAP) visualization of an output autoencoder embeddings.

**Figure 7:**
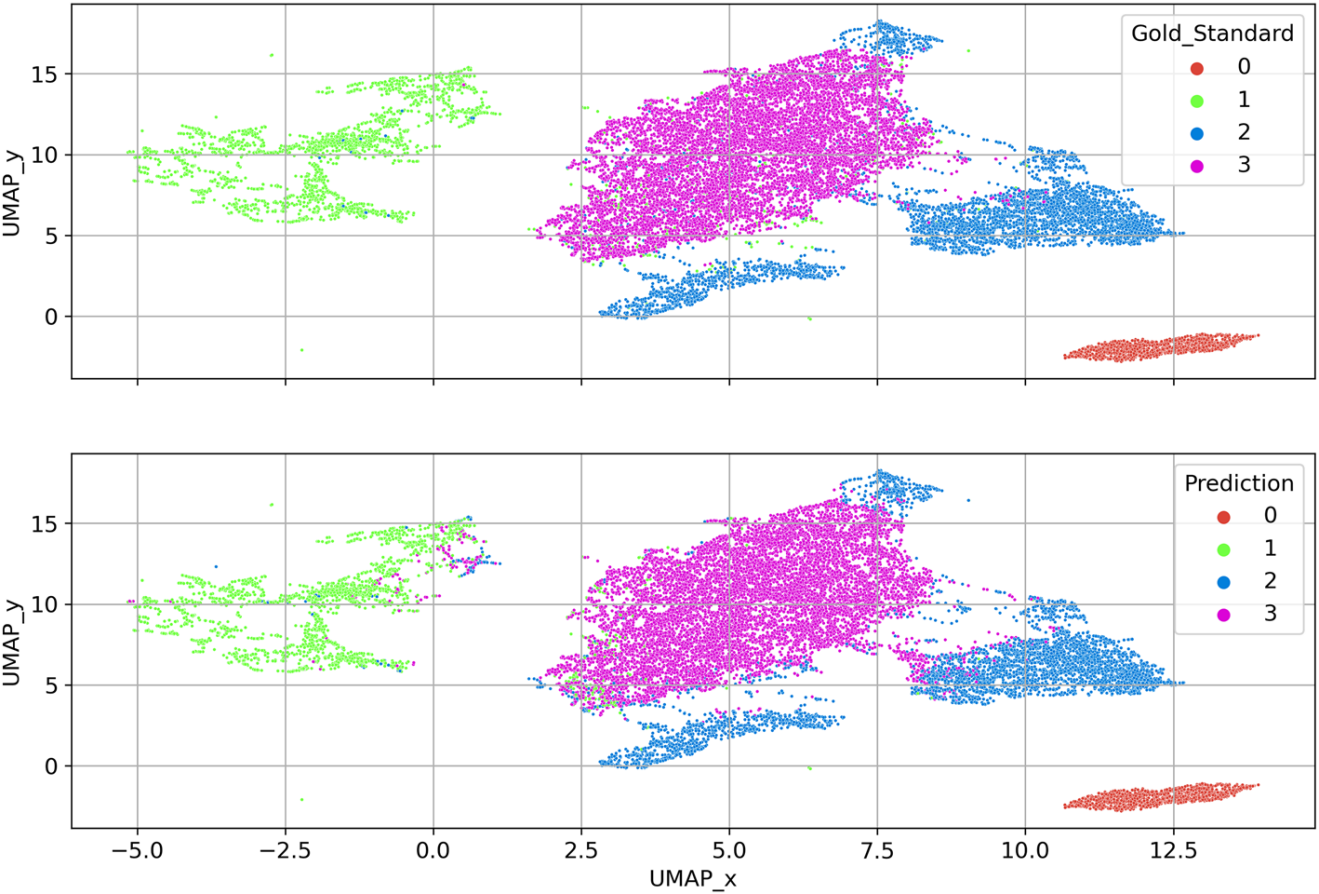
A representative example of uniform manifold approximation and projection (UMAP) of autoencoder embeddings from the testing set. The top picture depicts gold-standard data projections, and the bottom picture depicts predictions of the model. The shown model used 700 annotated examples in each classification category for training and achieved an F1-score of 0.91 evaluated on the out-of-sample test set. The classes are ordered as follows: 0-Power line noise, 1-Artifacts, 2-Pathological Activity, 3-Physiological Activity.

**Figure 8:**
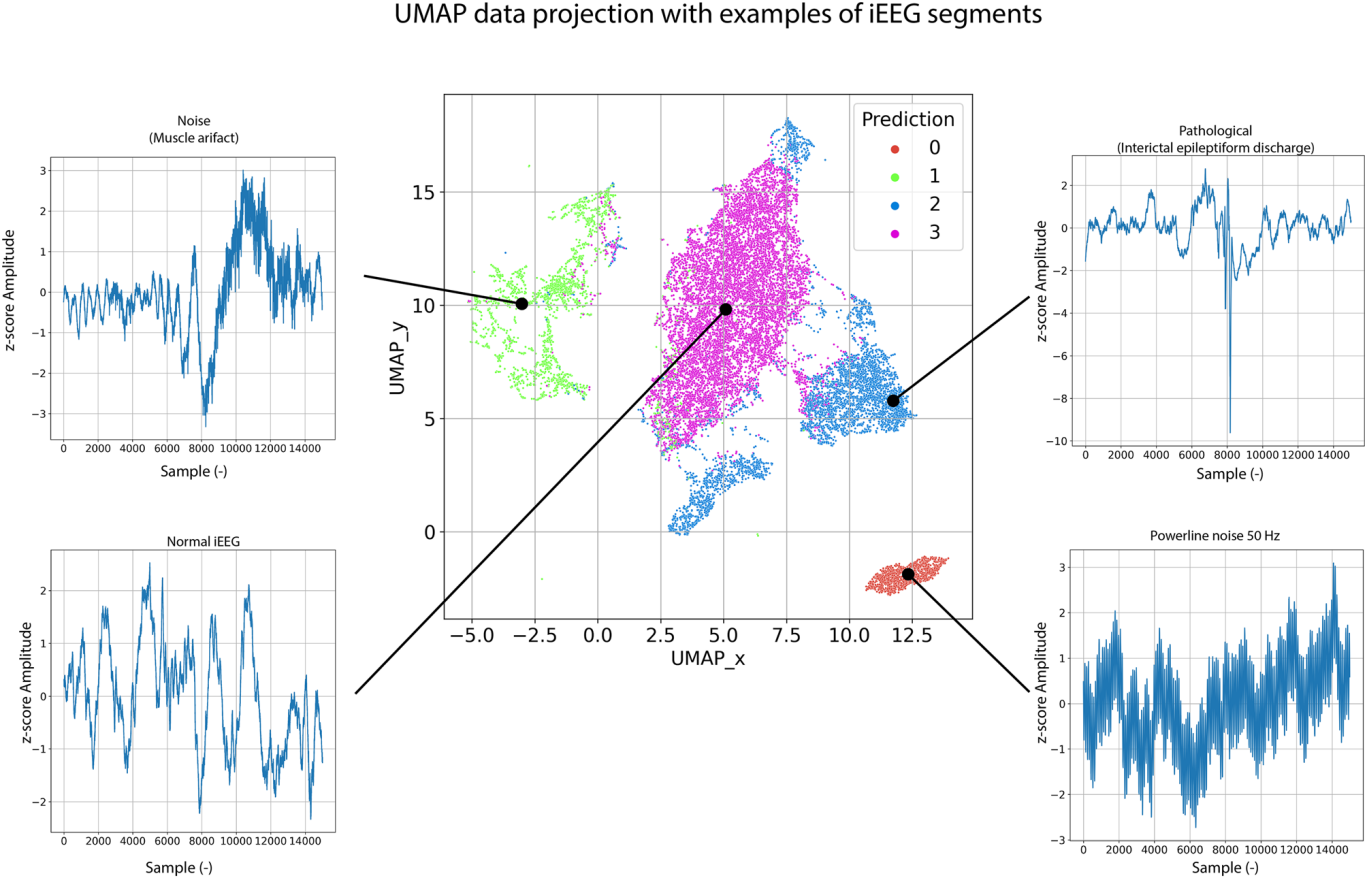
UMAP data projection with examples of iEEG segments from different clusters. The classes are ordered as follows: 0-Power line noise, 1-Artifacts, 2-Pathological Activity, 3-Physiological Activity.

To visually check whether the autoencoder provides reasonable embeddings (i.e,. similar iEEG segments should be closer to each other in the embedding space), we can further project embeddings into two-dimensional space (Figs. 6, 7, 8) using uniform manifold approximation and projection (UMAP). The testing set embeddings are UMAP projected and colored using its gold-standard (Fig. 7, top) and model predictions (Fig. 7, bottom).

### Ethics statement

This study was carried out in accordance with the approval of the Mayo Clinic Institutional Review Board with written informed consent from all subjects. The protocol was approved by the Mayo Clinic Institutional Review Board and St. Anne’s University Hospital Research Ethics Committee and the Ethics Committee of Masaryk University. All subjects gave written informed consent in accordance with the Declaration of Helsinki. All methods were performed in accordance with the relevant guidelines and regulations.

## Results

### Cross-validation results

**Figure 9:**
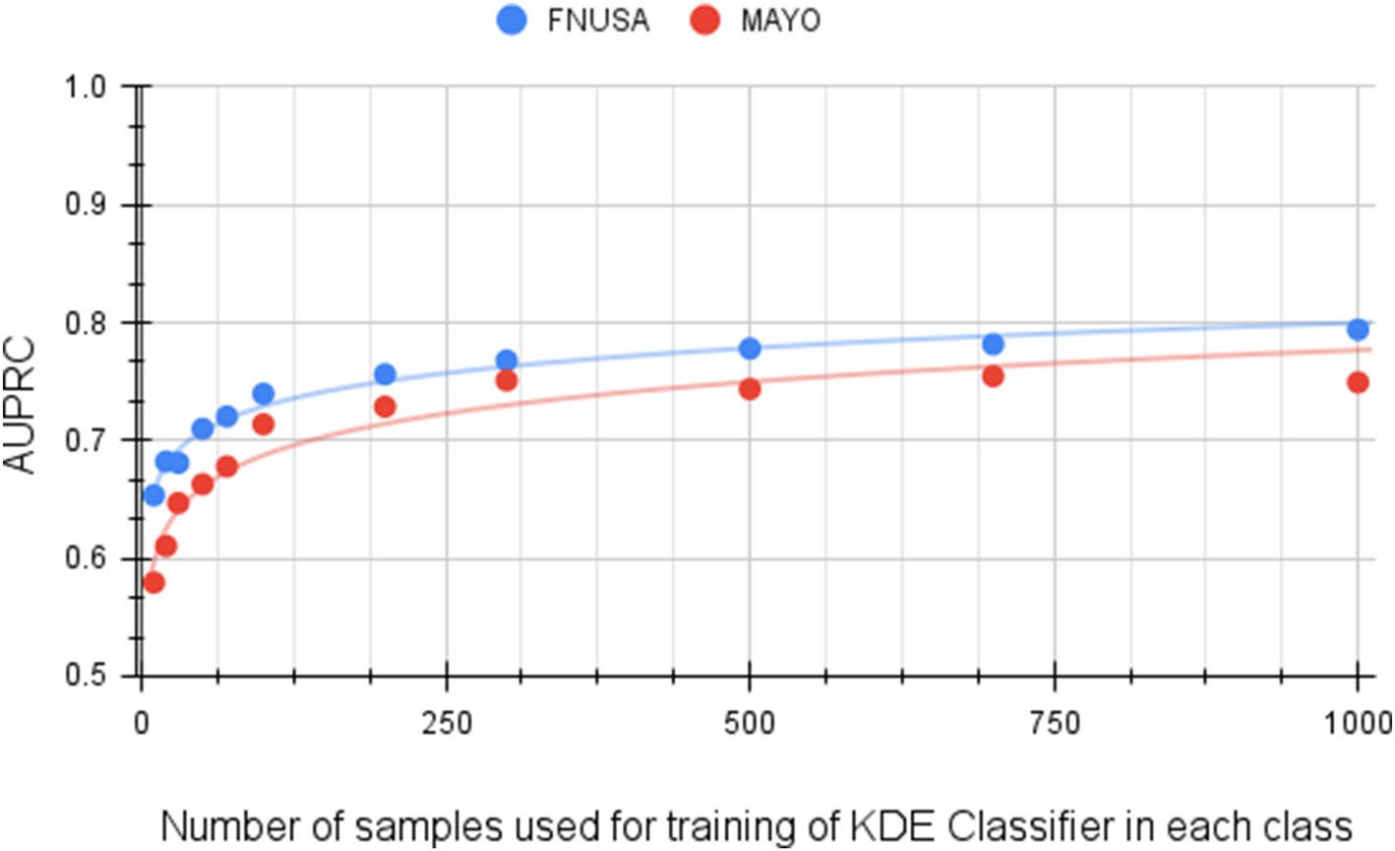
Performance of the model on two datasets related to the cardinality of training data for kernel density estimates (KDE) classifier. The plot shows the area under the precision-recall curve (AUPRC) metrics. The picture shows that 100 training examples per class achieve good model performance while requiring a reasonable amount of training labels.

We ran the model training for various cardinalities of labeled data from 10 to 1000 segments per category , and then the model classified the rest of the data from all 39 patients across two institutions. Table 4, shows results using the area under the receiver operating characteristic (AUROC) and the area under the precision-recall curve (AUPRC) as a quantification of the model performance (Table 4). Scores are given for all data in each institution. The scores were calculated as averages over all four training classes and 10 cross-validation batches. The values are written in the form avg ± std. Figure 9 shows that the model performs well (minimum AUROC 0.85 for FNUSA dataset) when at least 100 training samples are supplied to the KDE classifier as training data.

**Table 4:** Description of cross-validation results for FNUSA and MAYO datasets.

Segments per category	AUROC		AUPRC	
	FNUSA	MAYO	FNUSA	MAYO
1000	0.895 ± 0.036	0.896 ± 0.042	0.794 ± 0.071	0.749 ± 0.042
700	0.886 ± 0.039	0.898 ± 0.041	0.782 ± 0.075	0.755 ± 0.041
500	0.885 ± 0.031	0.893 ± 0.041	0.778 ± 0.064	0.744 ± 0.041
300	0.881 ± 0.036	0.896 ± 0.037	0.768 ± 0.066	0.751 ± 0.037
200	0.873 ± 0.030	0.886 ± 0.044	0.756 ± 0.060	0.729 ± 0.044
100	0.862 ± 0.037	0.879 ± 0.042	0.740 ± 0.066	0.714 ± 0.042
70	0.851 ± 0.032	0.861 ± 0.043	0.721 ± 0.053	0.678 ± 0.043
50	0.842 ± 0.031	0.855 ± 0.043	0.710 ± 0.056	0.663 ± 0.043
30	0.826 ± 0.025	0.849 ± 0.042	0.681 ± 0.039	0.647 ± 0.042
20	0.821 ± 0.036	0.825 ± 0.042	0.682 ± 0.057	0.611 ± 0.042
10	0.803 ± 0.021	0.806 ± 0.046	0.654 ± 0.033	0.580 ± 0.046

The table shows area under receiver operating curve (AUROC) and area under precision-recall curve (AUPRC).

### Pseudo-prospective testing for automated detection of Interictal Epileptiform Discharges

**Figure 10:**
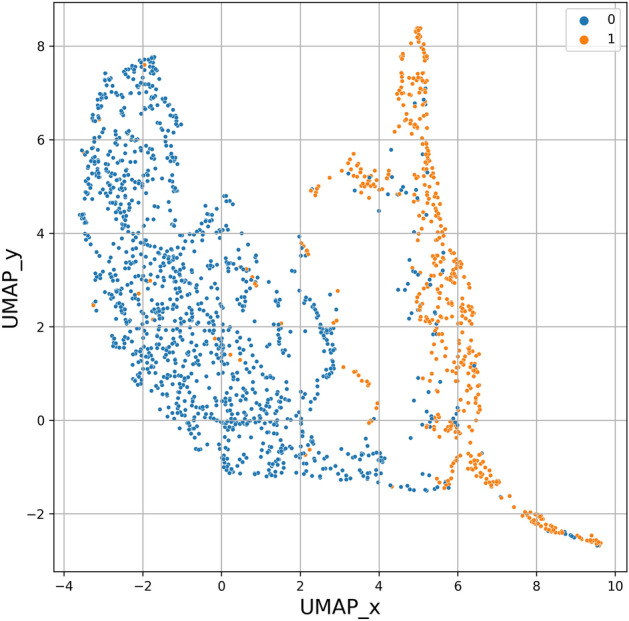
The figure shows a uniform manifold approximation and projection (UMAP) projection of a 30-min iEEG recording from a patient that was not included in the training, validation, or model testing process. The labels show gold-standard iEEG segments with 0-physiological iEEG, 1-interictal epileptiform discharges (IEDs). The separation between labels indicates that the autoencoder was able to generalize to novel data and cluster the data from two distinct categories to two minimally overlapping segments.

To show the power of the technique and its generalizability, we also tested the method's ability to detect pathological segments (i.e., IEDs) in novel data. We utilize novel data from 12 patients for a pseudo-prospective analysis (Table 1). An approximately 30-minute long iEEG recording from patients that were not included in the dataset for training and validation was manually verified, and every single IED was scored. Gold standards were made by a single expert. The pretrained model was deployed on this data, and data embeddings were subsequently projected into 2D space via UMAP to visualize the resulting data distribution. Visual inspection of Figure 10 reveals that most segments containing IED (orange dots) were clustered together, while physiological signals (blue dots) were clustered together. The cluster separation indicates that the autoencoder model could generalize to the novel data. The segments with IED have similar embeddings, and their distribution might be easily estimated by the pretrained KDE method. The results (Table 6) show AUROC and AUPRC scores.

## Discussion

In this work, we introduced a semi-supervised method for iEEG clustering and classification.The main purpose of the method is to enable objective and fast inspection of novel big electrophysiological data (presurgical evaluation of iEEG or long-term data from neurostimulator with sensing). The operator can use the method to generate data clusters with similar iEEG patterns (e.g., IEDs or artifacts) and then quickly label them to clinically useful categories quickly. The ultimate goal of the method is to make the annotation process objective and optimized for large-scale datasets and thus minimize the workload and time of an expert.

We propose a temporal context-aware method for semi-supervised classification in an active learning scenario using expert-in-the-loop. We applied the approach to heterogeneous multiscale electrophysiology data of two independent centers collected from 39 patients in hospitals across EU and the US. Furthermore, we present a method use case applied to IED detection where we tested the method pseudo-prospectively on 12 patients (Table 6, Fig. 10). We showed that the model was able to generalize to novel data. The model was able to cluster and differentiate IEDs from normal physiological iEEG in a 30-min long iEEG data segment and thus localize the data with IEDs. The presented method automatically processes one iEEG channel of 30-min recording in a few seconds. This enables substantially faster review of the data in comparison with the manual approach that can take tens of minutes (depending on the IED rate and capabilities of software used for EEG review).

The method utilizes data embeddings from the temporal autoencoder and visualizes it as a part of the expert-in-the-loop active learning process, where physicians subsequently label only a few members of each category (e.g., 100). Subsequently, the method estimates the KDE maps and trains the KDE classifier. We believe that this approach using the temporal context-aware autoencoder can be easily adopted in real-world scenarios where large amounts of unlabeled data are available and need to be reviewed. We tested the model on a previously published dataset of iEEG data. The model showed solid and reliable performance on datasets from both hospitals while using only 100 gold standard examples per class (Tables 4, 5, Figs. 7, 8).

The proposed approach achieved satisfactory results compared with fully supervised techniques previously presented in our study^20^ (Table 5). The two main benefits of the method are as follows. First, only a small number of labels can be used in comparison to the fully supervised techniques that require higher cardinality of the labeled data. Thus the method is less expensive. Second, the visual inspection of KDE maps can be used to suggest the best candidates for gold-standard scoring that will be subsequently used for training and optimization loops.

We believe that the proposed method might be efficiently used in the active learning expert-in-the-loop classification paradigm, where the expert is iteratively providing gold-standard labels that are automatically processed in order to generalize onto data points without labels. For example, this can be efficiently used for the detection of artifacts or epileptiform spikes in long-term recordings. The human expert can select a few examples, and the model automatically scans through the whole recording while marking similar data segments and suggesting other borderline and outlier samples in successive iterations for labeling by an expert to adapt to the changing nature of iEEG data. Clearly, in smaller datasets, this would save the time of the expert. In large datasets, it would enable the labeling of data that could not be reviewed at all.

In summary, the proposed method improves and accelerates review and annotation of large-scale datasets. In contrast with results of previously established works, we show that our system only requires 100 labeled instances of data per class to train the model that performs well (Table 5).

**Table 5:** Comparison of the proposed method with fully supervised state-of-the-art method.

Method	AUROC		AUPRC	
	FNUSA	MAYO	FNUSA	MAYO
Fully supervised Nejedly et al.^36^ (tens of thousands training samples)	0.92	0.97	0.80	0.93
Proposed method with 1000 KDE training samples per class	0.895	0.896	0.794	0.749
Proposed method with 100 KDE training samples per class	0.862	0.879	0.740	0.714

### Limitations

Our method was trained, validated and tested on iEEG data collected from patients with drug resistant epilepsy. The novel pseudo-prospective testing set was scored only by one expert iEEG scientist, under the supervision of an expert physiologist.

Currently, we can not infer the method's performance on scalp EEG, which in general has a large spectrum of neurological diseases. The generalization to scalp EEG would need follow-up study (Table 6).

**Table 6:** The table shows AUROC, AUPRC, and F1 results for the IED detection in pseudo-prospective testing of the proposed method.

Patient	AUROC	AUPRC	Macro F1 score	Weighted F1 score
1	0.866	0.623	0.77	0.93
2	0.843	0.619	0.71	0.80
3	0.964	0.88	0.90	0.96
4	0.919	0.863	0.88	0.90
5	0.909	0.708	0.81	0.96
6	0.816	0.67	0.76	0.83
7	0.948	0.901	0.91	0.92
8	0.738	0.396	0.68	0.87
9	0.833	0.701	0.55	0.54
10	0.93	0.856	0.84	0.9
11	0.803	0.57	0.71	0.8
12	0.887	0.84	0.82	0.82
MED	0.877	0.705	0.79	0.89
STD	0.067	0.154	0.11	0.12

## Conclusion

We proposed a semi-supervised method utilizing a temporal autoencoder for iEEG data classification. The proposed method achieved AUROC scores of 0.862 ± 0.037 and 0.879 ± 0.042 while using only 100 training examples per classification category scored by an expert. The method performed similarly in datasets from two different institutions, where iEEG recordings were captured with different acquisition systems and during different behavioral states (resting state and overnight Intensive Care Unit monitoring). We also tested the method pseudo-prospectively for IED detection, and the method achieved the AUROC of 0.877 ± 0.067 and AUPRC of 0.705 ± 0.154. Our results showed that the proposed method could massively shorten the time needed for manual ground-truth annotation. Therefore, it enables fast, efficient, unbiased data exploration in heterogeneous time-series large-scale datasets (e.g., clinical research settings).

## Data Availability

The datasets used for model training and cross-validation are publicly available for download at figshare repository (https://doi.org/10.6084/m9.figshare.c.4681208). The information about data might be obtained from our data descriptor paper^36^.
